# The olivo-cerebellar system and its relationship to survival circuits

**DOI:** 10.3389/fncir.2013.00072

**Published:** 2013-04-23

**Authors:** Thomas C. Watson, Stella Koutsikou, Nadia L. Cerminara, Charlotte R. Flavell, Jonathan J. Crook, Bridget M. Lumb, Richard Apps

**Affiliations:** ^1^School of Physiology and Pharmacology, Medical Sciences Building, University of Bristol, University WalkBristol, UK; ^2^Queensland Brain Institute, The University of QueenslandBrisbane, QLD, Australia

**Keywords:** cerebellum, inferior olive, periaqueductal gray, survival, modules

## Abstract

How does the cerebellum, the brain’s largest sensorimotor structure, contribute to complex behaviors essential to survival? While we know much about the role of limbic and closely associated brainstem structures in relation to a variety of emotional, sensory, or motivational stimuli, we know very little about how these circuits interact with the cerebellum to generate appropriate patterns of behavioral response. Here we focus on evidence suggesting that the olivo-cerebellar system may link to survival networks via interactions with the midbrain periaqueductal gray, a structure with a well known role in expression of survival responses. As a result of this interaction we argue that, in addition to important roles in motor control, the inferior olive, and related olivo-cortico-nuclear circuits, should be considered part of a larger network of brain structures involved in coordinating survival behavior through the selective relaying of “teaching signals” arising from higher centers associated with emotional behaviors.

## INTRODUCTION

A neural network of structures including, but not confined to, components of the limbic system (e.g., prefrontal cortex, amygdala, and hypothalamus) and closely linked brainstem structures (e.g., periaqueductal gray, PAG), are known to play a critical role in coordinating functions essential for survival, including a variety of emotionally related defensive behaviors triggered by aversive (e.g., fearful) or painful events (Bandler et al., 2000; Sokolowski and Corbin, 2012). Historically, considerable attention has been devoted to mapping activity within different components of these “survival circuits” in relation to a variety of sensory, emotional, or motivational stimuli (cf. LeDoux, 2012). In marked contrast, we know much less about how these circuits interact with the motor system to generate appropriate patterns of behavioral response. The aim therefore of this short review is to discuss evidence, including recent observations, which together suggest that the concept of survival circuits should be extended to include the olivo-cerebellar system. In particular, we will focus on cerebellar interactions with the PAG; a structure with a well characterized role in survival behaviors.

## PAG AND SURVIVAL

The PAG is generally accepted to be a pivotal component of a central “survival network”. It is a behaviorally important source of descending control that is activated in response to a variety of emotional and environmental stressors, such as fear, anxiety, and pain (Bandler et al., 1991), and is crucial in controlling the expression and co-ordination of responses in these contexts (Fanselow et al., 1991; Carrive et al., 1997; Walker and Carrive, 2003). These controls include cardiovascular regulation, sensory modulation and the generation of a variety of emotionally related motor behaviors, such as fight/flight or immobility/withdrawal from the environment (commonly known as *active* and *passive* coping, respectively).

Active coping enables an animal to escape a stressor (e.g., brief acute pain or encounter with a predator), and is elicited from a column of neurons situated in the dorsolateral/lateral (dl/l) functional column of the PAG. Activation of dl/lPAG increases arterial blood pressure, increases mobility (fight-or-flight responses) and elicits characteristic defense postures, e.g., the animal displays “reactive immobility” in that it is tense and ready for action but is temporarily motionless (Carrive, 1993; Lovick, 1993; Bandler and Shipley, 1994; Fendt and Fanselow, 1999; Keay and Bandler, 2001; Lumb and Leith, 2007). The dl/lPAG can be further divided into rostral and caudal segments with distinct defensive responses associated with upper and whole body movements, respectively (Bandler et al., 1991). By contrast, passive coping is characterized by a general disengagement from the environment when a stressor is inescapable (e.g., chronic pain) or when evading detection during close encounter with a predator. Passive coping is coordinated by a column of neurons located in ventrolateral (vl) PAG and is associated with a reduced responsiveness to external stimuli, and a general cessation in movements and a fixed (freezing) posture (Zhang et al., 1990; Bandler et al., 1991; Carrive, 1993; Lovick, 1993; Bandler and Keay, 1996). As part of these complex coping strategies, the PAG exerts descending control of spinal sensory processing that not only discriminates between noxious and non-noxious events but also between nociceptive inputs of different behavioral significance; C-nociceptor-evoked activity (mediating the slowly conducted, poorly localized and therefore distracting component of the nociceptive message) is depressed while A-nociceptor-evoked activity (the rapidly conducted component that encodes the intensity of the nociceptive signal; McMullan and Lumb, 2006b) is left intact or even enhanced. Indeed, previous studies indicate that this pattern of effects could operate as part of both active and passive coping strategies that are co-ordinated by the dl/l- and vl-PAG, respectively. Therefore, in both situations differential control of A- vs C-fiber-evoked activity could preserve the detailed information of changes in the external environment that can drive motivational behaviors and accurately direct motor activity (A-fibers), whilst depressing those components of the nociceptive message (C-fibers) that are less useful in terms of survival (e.g., enabling escape behavior without the distraction of C-fiber mediated pain; Waters and Lumb, 1997; McMullan and Lumb, 2006a,b; Koutsikou et al., 2007; Heinricher et al., 2009; Leith et al., 2010).

In summary, outputs from the different functional columns in the PAG co-ordinate fundamentally different patterns of autonomic adjustment, sensory regulation and motor responses that are highly dependent on the behavioral significance of the environmental, emotional or sensory stimulus.

In terms of PAG function, attention to date has focused on neural pathways that underlie autonomic regulation and sensory control, and polysynaptic descending paths that modulate autonomic outflow and sensory processing at the level of the spinal cord are well described (Lovick and Bandler, 2005). In contrast, much less is known about the neural pathways and mechanisms that link PAG activity to distinct patterns of motor responses. Until recently (Cerminara et al., 2009; see below) we knew very little about whether descending control extends to sensory signals that feed into (and can modify) supraspinal motor circuits that co-ordinate movement. Furthermore, scant information is available on how sensorimotor structures, such as the cerebellum, can in turn, modulate activity within the PAG.

## THE CEREBELLUM AND MODULAR ORGANIZATION

The cerebellum is involved in regulating a wide range of brain functions including autonomic and somatic reflexes, and voluntary movements (Ito, 1984). Recent neuroanatomical tracing, lesion and neuroimaging studies suggest that the cerebellum may also be involved in higher order processes (Schmahmann, 2004; Strick et al., 2009) including emotional behaviors. Anatomically and functionally, the cerebellum can be subdivided into a series of units termed “modules” that are highly conserved across mammalian species (Apps and Hawkes, 2009). Structurally, each module is defined by a specific climbing fiber input from a discrete part of the inferior olivary complex, which targets one or more longitudinal zones of Purkinje cells within the cerebellar cortex. In turn, the Purkinje cells within each zone project to a specific region of the cerebellar and vestibular nuclei (which themselves receive axon collaterals from the same olivary cells). Since all cerebellar cortical processing is forwarded to the cerebellar nuclei, the latter ultimately control cerebellar contributions to behavior. Cerebellar nuclear output is mainly excitatory (Batini et al., 1992) and projection neurons exert a powerful modulatory influence on a variety of ascending and descending pathways; including brainstem structures associated with the survival network (Ito, 1984; Armstrong, 1986).

Physiologically, olivo-cerebellar inputs, mediated by climbing fibers, are considered central (but not exclusive) to theories of cerebellar-dependent learning (Marr, 1969; Albus, 1971; Ito, 1972). In brief, it is thought that climbing fiber input acts as a teaching signal, which triggers plastic changes in synaptic efficacy in the cerebellar cortex (namely, long-term depression of parallel fiber synaptic transmission). Furthermore, these teaching signals are regulated or “gated” in a task dependent manner and it has been hypothesized that this ensures the transmission of only behaviorally relevant training signals (for review see Apps, 1999, 2000). However, currently we know relatively little about how gating relates to teaching signals arising from higher brain structures, including those involved in the survival network.

Many regard olivo-cortico-nuclear modules as a fundamental feature of cerebellar contributions to motor control and indeed many other functions such as cognition and autonomic regulation (Yeo and Hesslow, 1998; Nisimaru, 2004; Apps and Garwicz, 2005; Ramnani, 2006). Of particular relevance to the suggestion that the cerebellum should be considered part of a distributed survival network is the growing body of evidence that the cerebellar vermis (including the A module and associated output nucleus, fastigius), which has an established role in the regulation of posture, balance (e.g., Cerminara and Apps, 2011) and oculomotor control (Voogd and Barmack, 2006), also serves as a critical component of a network subserving emotionally related behaviors (Strick et al., 2009; Strata et al., 2011). Indeed, Sacchetti et al. (2004) have shown that brief, reversible tetrodotoxin (TTX) inactivation of vermis lobules V and VI impairs consolidation of fear memories and that cerebellar long-term synaptic plasticity is potentiated in fear-conditioned animals. Sacchetti and colleagues (2007) have also provided evidence that the cerebellar vermis (lobules V and VI) supports fear memory processing in the absence of the amygdala (the latter is generally regarded as a central component of the survival network). Collectively, these findings therefore suggest that the cerebellum, like the amygdala, is involved in the processing of fear related memory and associated defensive behaviors. However, the precise role of individual olivo-cortico-nuclear modules in survival networks remains to be established.

## EVIDENCE OF A PAG – CEREBELLAR LINK

**FIGURE 1:**
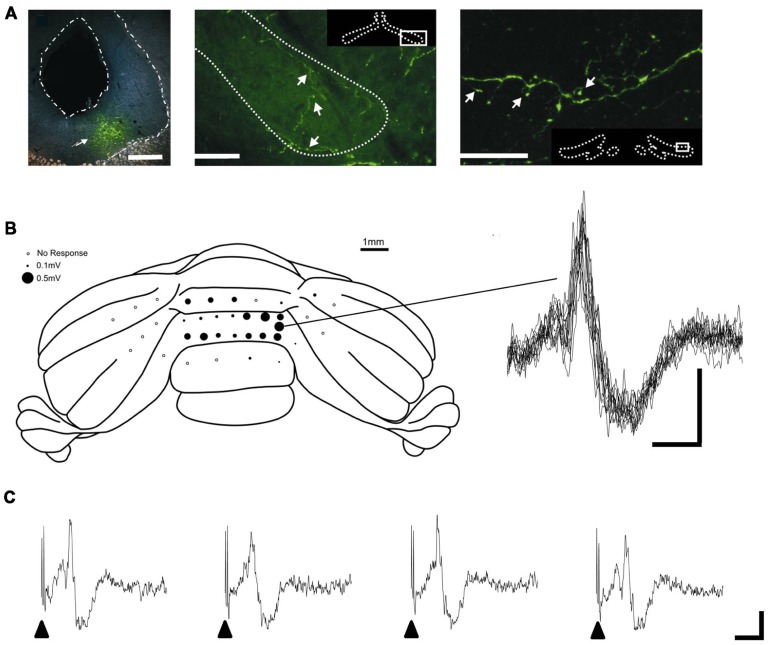
**PAG-olivo-cerebellar connectivity**. **(A)** Microinjection of viral anterograde tracer (AAV-CMV-eGFP) into the vlPAG (left panel, injection site indicated by arrowhead; scale bar, 0.5 mm) leads to terminal labeling in the medial accessory olive (middle panel, indicated by arrowheads; scale bar, 100 μm) and dorsal accessory olive (right panel, indicated by arrowheads; scale bar, 30 μm) and also principal olive (not shown). **(B)** Left, posterior view of the rat cerebellum illustrating the distribution of responses evoked by stimulation of left dlPAG at intensity of 2x threshold in one experiment under sodium pentobarbital anasthesia (60 mg/kg administered intraperitoneally). Mean peak-to-trough amplitude (three trials) is represented by the size of filled circles. Right, example waveforms (superimposition of eight consecutive responses) obtained from the recording position indicated on the cerebellar schematic. Stimulus delivered at start of each trace. Scale bar, 15 ms and 0.2 mV. **(C)** Example of four consecutive responses recorded from the same position on the cerebellar cortex. Filled arrow heads indicate timing of PAG stimulation. Scale bars, 20 ms and 0.2 mV. Reproduced with permission from Flavell (2008) and Crook et al., unpublished.

Given the key role of the PAG in survival circuits, interactions with the cerebellum may provide an important mechanism through which co-ordinated movements can be modulated to enhance survival behaviors in aversive or threatening situations. Anatomical mapping studies provide at least some evidence that interconnections exist between the PAG and cerebellum. Direct, bilateral projections from vlPAG to the cerebellar cortex were first described by Dietrichs (1983). The diffuse nature of the projection suggests the pathway most likely terminates as mossy fibers. In addition, several lines of anatomical evidence suggest that the PAG has links with the cerebellum via the inferior olive – climbing fiber system. Several studies have noted the presence of an ipsilateral projection from the PAG to the olive, including the caudal medial accessory olive (cMAO, Rutherford et al., 1984; Holstege, 1988). This region of the olive provides climbing fiber projections to the cerebellar vermis (Apps, 1990). The presence of such connectivity has been confirmed by using modern viral vector tracer techniques, which have the advantage over conventional tracers in that the results are not confounded by tracer uptake by axons of passage (Flavell, 2008). In brief, by using targeted microinjections of green fluorescent protein (GFP) tagged adeno-associated virus-cytomegalovirus-enhanced GFP (AAV-CMV-eGFP) into vlPAG, Flavell, 2008 demonstrated a widespread but diffuse projection to all major subdivisions of the olive (see **Figure 1A**). Electrophysiological mapping studies have also shown that microstimulation in dorsal PAG elicits large field potentials localized to cerebellar vermis lobules VII/VIII – which have well defined roles in the control of oculomotor and cardiovascular functions (Noda and Fujikado, 1987; Nisimaru, 2004; Voogd and Barmack, 2006) – with a mean onset latency of 15.2 ± 0.8 ms (*n* = 5 rats, three trials per rat; Crook et al., unpublished observations; see **Figures 1B,C**). The waveform and trial-by-trial fluctuations in size of these evoked field potentials are typical of climbing fiber mediated responses (Armstrong and Harvey, 1968).

What role might the PAG link with the olivo-cerebellar system serve? In attempting to address this question there are two points worth noting. First, climbing fiber afferents, which terminate in a range of cerebellar cortical zones, are powerfully activated by nociceptive inputs (Ekerot et al., 1987). Second, climbing fiber pathways originating from the spinal cord (spino-olivocerebellar paths, SOCPs) are subject to central modulation during motor learning and active movements (Apps, 1999). Given the well known role of the PAG in regulating transmission of nociceptive signals at the level of the spinal cord, this raises the possibility that the link with the olivo-cerebellar system serves a similar function.

**FIGURE 2:**
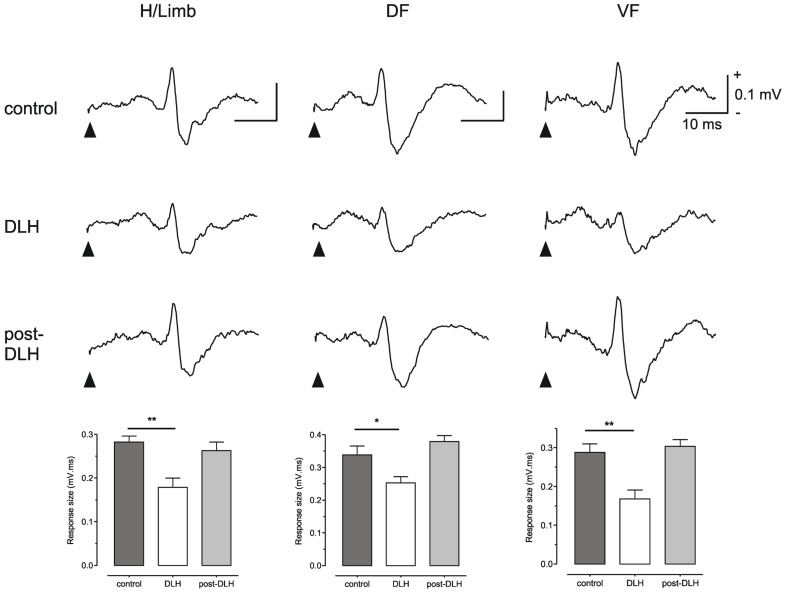
**The PAG can modulate SOCPs supraspinally**. Climbing fiber fields evoked by stimulation of contralateral hindlimb (H/Limb), ipsilateral dorsal funiculus (DF) and contralateral ventral funiculus (VF) are all significantly suppressed following microinjection of an excitatory amino acid (D-homocysteic acid, DLH) into the vlPAG. Stimulation onset indicated by filled arrowheads. Each trace is an average of 15 trials. Each bar is the mean + SEM of *n* = 15 trials. *P < 0.05; **P < 0.01. Scale bar values are the same for each trace. Reproduced with permission from Cerminara et al. (2009).

To test this possibility Cerminara and colleagues (2009) electrically stimulated the hindlimb and recorded climbing fiber field potentials in the C1 zone of the ipsilateral copula pyramidis of anesthetized rats, and found that the size of the evoked cerebellar responses (generated as a result of transmission in SOCPs) could be significantly reduced by chemical neuronal activation of vlPAG (**Figure 2**). The climbing fiber responses evoked in this region of the cerebellar cortex are relayed by two SOCPs; one conveys ascending signals via the dorsal funiculus, the other via the ventral funiculus (Oscarsson, 1969; Armstrong et al., 1973; Oscarsson and Sjolund, 1977a,b,c). Importantly, responses evoked by electrical stimulation of the dorsal or ventral funiculus were also reduced by PAG activation (**Figure 2**). This demonstrates that modulation of SOCPs by the PAG must, at least in part, occur supraspinally. Since the ventral funiculus has direct projections to the inferior olive (Boesten and Voogd, 1975; Oscarsson and Sjolund, 1977a,b,c) this finding is consistent with the proposal that the PAG regulates transmission of ascending sensory signals at the level of the olive.

Direct anatomical projections from the PAG to the olive may have a role in this control, but this of course does not exclude the possibility that other (indirect) pathways are also involved. Descending connections to the PAG from higher structures such as the prefrontal cortex (Beitz, 1982; Keay and Bandler, 2001), may also be a route through which neocortical centers that are involved in emotionally related behavior can gain access to the olivo-cerebellar system. The finding that electrical stimulation of the prelimbic subdivision of rat prefrontal cortex powerfully drives activity in olivo-cerebellar pathways supports this hypothesis (Watson et al., 2009).

## CEREBELLAR OUTPUT TO SURVIVAL CIRCUITS

Important insights into cerebellar contributions to survival circuits can also be gained from anatomical/physiological analysis of cerebellar output (cf. Strick et al., 2009). In particular, several lines of evidence suggest the cerebellar fastigial nucleus has links with limbic structures involved in survival behaviors, such as the hippocampus, hypothalamus, ventral tegmental area (VTA), and amygdala (e.g., Snider and Maiti, 1976; Newman and Reza, 1979; Cao et al., 2013). In respect to cerebellar-PAG projections, Whiteside and Snider (1953) showed that electrical stimulation of vermal lobule VII in the anesthetized cat can evoke responses in the dorsal PAG with two distinct latencies (2–3 ms and 8–12 ms), which raises the possibility that multiple cerebello-PAG pathways exist. Consistent with a direct (short latency) projection, anatomical tracing studies have shown the existence of efferent fastigial projections to the PAG in a number of species (Martin et al. (1974) in the opossum; Beitz (1982); Gonzalo-Ruiz and Leichnetz (1987), Gonzalo-Ruiz et al. (1990) and Teune et al. (2000) in the rat, and Gonzalo-Ruiz et al. (1988) in monkey). Many of these studies have advanced the view that the projections subserve an oculomotor function. However, it is possible that functions of fastigial-PAG projections are more wide ranging and enable the powerful computational circuitry of the cerebellum to engage with circuits related to the expression of survival behaviors. Consistent with this proposal, clinical studies have shown that chronic stimulation of the cerebellar vermis can be used to regulate emotion and “correct behavior” in human patients suffering from intractable neurological disorders such as schizophrenia and epilepsy (Cooper, 1973a,b; Cooper et al., 1973a,b; Correa et al., 1980; Heath et al., 1980a,b,^1981^). Furthermore, transmagnetic stimulation of the medial cerebellum in humans can also provide anti-depressive effects (Schutter and van Honk, 2005,^2009^; Hoppenbrouwers et al., 2008; Schutter et al., 2009a,b) and can enhance the power of neuronal oscillations, within the theta and gamma frequency range, across regions of the frontal cortex that are thought to be essential to cognitive and emotional aspects of behavior (Schutter et al., 2003; Schutter and van Honk, 2005).

In experimental animals, lesion of the fastigius has a wide variety of behavioral effects such as drowsiness (Fadiga et al., 1968; Giannazzo et al., 1968a,b; Manzoni et al., 1968), aggression (Reis et al., 1973), and grooming behavior (Berntson et al., 1973; Reis et al., 1973). In addition, reductions in activity such as open-field exploratory behavior, and social interactions, independent of non-specific motor abnormalities, have been demonstrated following fastigial lesions in rat (Berntson and Schumacher, 1980). Finally, stimulation of the cerebellar vermis or the fastigial nucleus can elicit a variety of complex patterns of defense-like behavior such as sham rage and predatory attack (Zanchetti and Zoccolini, 1954; Reis et al., 1973). Given the links with PAG and other components of the survival network it seems reasonable to infer that the diverse effects of cerebellar manipulations are in part due to its interactions with survival circuits. The fact that the fastigial nucleus is the output for several cerebellar modules (A, AX, A2) raises the possibility that the range of different behaviors reported in the literature may be due to differential activation of one or more of these pathways.

## CONCLUDING COMMENTS

The aim of this short review has not been to consider cerebellar interactions with every structure in the survival network; rather we have focused specifically on the cerebellar-PAG link as an illustrative example. Overall, the available neuroanatomical and physiological evidence suggests that the necessary interconnectivity exists to consider the inferior olive and cerebellum as additional components of a distributed “survival behavior network”. The functional significance of olivo-cerebellar involvement in this network remains to be determined, but one influential theory of climbing fiber function is that they serve a teaching role (for a review see for example Yeo and Hesslow, 1998). The powerful climbing fiber mediated projection from PAG to the cerebellar vermis and gating of SOCPs by the PAG may be considered in relation to this theory. Under appropriate behavioral conditions in which survival circuits are engaged, the gating may reflect a switch from the usefulness of learning signals derived from the periphery, to allowing signals arising from higher centers to modify cerebellar function.

## Conflict of Interest Statement

The authors declare that the research was conducted in the absence of any commercial or financial relationships that could be construed as a potential conflict of interest.
